# Absence of potential gadolinium toxicity symptoms following 22,897 gadoteric acid (Dotarem®) examinations, including 3,209 performed on renally insufficient individuals

**DOI:** 10.1007/s00330-018-5737-z

**Published:** 2018-10-01

**Authors:** Laura K. Young, Shona Z. Matthew, J. Graeme Houston

**Affiliations:** 1 Division of Molecular and Clinical Medicine, School of Medicine, University of Dundee, Ninewells Hospital and Medical School, Dundee, DD1 9SY UK; 2 Clinical Radiology, NHS Tayside, Ninewells Hospital and Medical School, Dundee, UK

**Keywords:** Magnetic resonance imaging,, Medical record linkage,, Contrast media,, Renal insufficiency

## Abstract

**Objectives:**

Recent safety concerns regarding gadolinium-based contrast agents (GdCAs) concluded with the suspension of some agents from the European market, yet a clinical consequence remains unknown. We used electronic health records to investigate the incidence of potential toxicity to gadoteric acid (Dotarem®) within our local population, including those with renal insufficiency (RI).

**Methods:**

Data for patients who underwent contrast-enhanced MRI were identified, stratified by renal function at time of scan and retrospectively followed using routinely collected health data. Searches performed were: records of hypersensitivity reactions; diagnoses of nephrogenic systemic fibrosis (NSF); onset of chronic pain, a symptom that has been associated with NSF and the theorised gadolinium deposition disease (GDD); and post-contrast acute kidney injury (PC-AKI). Comparisons were made between patients and controls (those who underwent non-contrast scans) via chi-square and ANOVA statistical tests.

**Results:**

Of the 22,897 contrast-enhanced MRI scans performed locally from 2004–2016 (adult, n = 22,325 and paediatric, n = 572), 14% were performed on patients with RI (30 ≤ eGFR < 60, n = 2,622; 15 ≤ eGFR < 30, n = 464; eGFR < 15, n = 123). Two adult patients (0.01%) suffered hypersensitivity reactions. Zero cases of NSF were reported, with an average follow-up time of 6.0 ± 2.5 years (range, 8 months–15 years). Analysis failed to highlight statistically higher rates of chronic pain onset post-MRI (adult: *p* = 0.777, paediatric: *p* = 0.578), or PC-AKI (adult: *p* = 0.566, paediatric: *p* = 0.841), in the patient groups compared to controls.

**Conclusions:**

These data indicate that administration of gadoteric acid to RI patients does not result in a higher rate of signs or symptoms that may be associated with gadolinium toxicity when compared to controls.

**Key Points:**

*• Following 22,897 administrations of gadoteric acid to a local population, there was no association with symptoms that may be associated with gadolinium toxicity.*

*• Zero cases of nephrogenic systemic fibrosis were reported following 3,209 gadoteric acid administrations to a cohort of renally insufficient patients.*

*• A low number of hypersensitivity reactions were observed (0.01%) and no higher rate of chronic pain or post-contrast acute kidney injury were noted when compared with a control cohort of non-contrast-enhanced examinations.*

## Introduction

Macrocyclic and linear gadolinium-based contrast agents (GdCAs) are routinely used in magnetic resonance imaging (MRI) to speed imaging and aid diagnosis. The stability of these agents varies, with the macrocyclic, ionic agents considered the most thermodynamically and kinetically stable, while linear, non-ionic agents, are considered less so [1]. In general, GdCAs have excellent safety profiles with immediate hypersensitivity reactions reported as between 0.01% and 2.4% [2, 3], yet the longer term safety of these agents has recently come under intense scrutiny. The deposition of gadolinium within the body following exposure to these agents has been proven [4–6], and appears to be dependent on factors such as agent stability and patient renal function. Whilst no clinical consequence has been confirmed, the term gadolinium toxicity has been ascribed to a broad range of likely pathologies [7].

Nephrogenic systemic fibrosis (NSF) was definitively linked to the administration of some GdCAs in patients with renal insufficiency (RI) in 2006 [8, 9]. This incurable skin-fibrosing condition, often accompanied with chronic pain [10], typically arises within 2–10 weeks [11] of contrast administration, and is seen with greater frequency in patients with more severe RI at the time of exposure. NSF was found to be directly related to the stability of GdCAs and, as such, in 2010 the European Medicines Agency (EMA) [12] and US Food and Drug Administration (FDA) [13] established guidelines and classified the agents based on their risk of inducing NSF. Post-contrast acute kidney injury (PC-AKI) is a sudden deterioration in renal function post-contrast media administration in the absence of other nephrotoxic events [14] and, although the development of PC-AKI following exposure to GdCAs remains highly disputed due to inconsistent study protocols, there is evidence to suggest that GdCAs can induce PC-AKI [15–17].

More recently, gadolinium has been found to accumulate in the body regardless of renal function [4, 18, 19]. The highest concentrations of gadolinium have been seen following exposure to the least thermodynamically stable GdCAs [6, 20, 21] and total concentrations within the CNS display an inverse correlation with renal function [22]. Following an extensive review, the EMA has ‘confirmed recommendations to restrict the use of some linear gadolinium agents used in MRI body scans and suspend the authorisation of others’ [23]. Clinical consequences of a theorised gadolinium deposition disease (GDD) have not yet been confirmed but case reports and published support group surveys suggest symptoms could be reminiscent of NSF [24–26]. The development of symptoms such as headaches, bone/joint pain and skin changes appear to occur earlier, with typical onset times reported between hours and days post-contrast-enhanced MRI [24, 25].

As the renal pathway is the predominant excretion mechanism of most GdCAs, patients with RI are considered at high risk of the above signs and symptoms of gadolinium toxicity and therefore a population of great interest. However, due to changes in guidelines and clinical practice, the use of GdCAs in populations with RI has declined dramatically [27, 28], and instigating contrast-enhanced MRI clinical trials would be challenging. In cases where the diagnostic information is unavailable via non-contrast images and the clinical benefit-to-risk ratio is favourable, low risk macrocyclic GdCAs have continued to be administered.

Locally, patient-specific electronic health records have been collated over many years, and as such, the aim of this study was to use this data in a unique way to follow adult and paediatric patients, particularly focussing on those with RI, who have been exposed to gadoteric acid (Dotarem®, Guerbet) in order to identify any hypersensitivity reactions or symptoms that may be associated with gadolinium toxicity such as NSF, PC-AKI and/or chronic pain.

## Materials and methods

### Ethical approvals

Approval for this work was granted by the East of Scotland Research Ethics Committee under the collective ReDVA project (a European Union’s 7th Framework Program grant agreement no. 324487). Whilst the routinely collected NHS patient data used for this study is not patient-consented, the research is approved by NHS Caldicott Guardian for anonymised analysis within the SafeHaven secure IT environment.

### Data

Local electronic patient data were used to identify and compile patient timelines. Patient data for those who had undergone solely gadoteric acid-enhanced scans were included into the study cohort. Data from those patients who had undergone solely non-contrast MRI were included as a control cohort. Accident & Emergency (A&E) admissions, biochemistry records (serum creatinine), dermatology and medication prescriptions were then gathered and linked for the entire study population.

Demography data such as gender and date of birth were provided for each patient in the study cohort. In combination with the examination date, age at scan was calculated. Renal function was described by serum creatinine for paediatric patients (0–17 years), while for adults (18+ years) serum creatinine was converted to estimated glomerular filtration rate (eGFR) via the Modification of Diet in Renal Disease (MDRD) study equation. Data collection was performed by a qualified data analyst, and following anonymisation, data linkage was performed by an experienced medical researcher using Microsoft Excel (2013 version, Microsoft) within the SafeHaven secure IT environment.

### Patient data

In total, the electronic records for 40,411 MRI scans and corresponding biochemistry, dermatology and prescription data were gathered and divided into contrast and non-contrast MRI. 1,333 scans pertaining to 947 patients were excluded due to documented use of GdCAs other than gadoteric acid. Within the local area between 2004 and 2016, 22,325 contrast-enhanced MRI were performed on 15,377 adult patients (average age, 55.6 ± 16.1 years; range, 18–97 years; 53.3% female) and 572 contrast-enhanced scans were performed on 370 paediatric patients (average age, 11.4 ± 5.3 years; range, 0–17 years; 51.4% female). Further demographic data are outlined in Table 1.

**Table 1:** Characteristics of patients undergoing MRI scans

Adults n = 37,813	Gadoteric Acid n = 22,325 Renal function stage				Non-contrast n=15,488 Renal function stage			
	1/2	3	4	5	1/2	3	4	5
No. of scans (n)	19,168	2,570	464	123	13,647	1,731	76	34
Age at first scan^**^ (y)	53.3 ± 15.4^a,b^ (18–97)	69.9 ± 11.6^a,b^ (20–97)	73.1 ± 12.7^a^ (19–93)	60.5 ± 15.3^a^ (29–88)	50.3 ± 16.0^a,b^ (18–94)	66.2 ± 13.5^a,b^ (19–94)	72.7 ± 13.2^a^ (32–89)	54.7 ± 17.3^a^ (28–81)
% Female	53.1^b^	53.5^b^	59.8	44.9	56.6^b^	70.4^a,b^	63.8	63.2
Dose per scan^**^(ml)	13.7 (5–45)	16.7 (5–45)	20.1 (5–45)	18.0 (10–40)	N/A	N/A	N/A	N/A
Cumulative dose per patient^**^ (mmol Gd)	10.3 (2.5–90.3)	11.8 (2.5–90.3)	12.8 (5.0–45.0)	13.0 (5.0–45.0)	N/A	N/A	N/A	N/A
Paediatrics n=1,265	Gadoteric acid n = 572 Renal function		Non-contrast n=693 Renal function					
	Normal	Insufficient	Normal	Insufficient				
No. of scans (n)	520	52	528	165				
Age at first scan^**^ (y)	11.6 ± 5.4^a,b^ (0–17)	9.7 ± 6.5^a,b^ (0–17)	9.6 ± 6.5^a,b^ (0–17)	7.6 ± 5.7^a,b^ (0–17)				
% Female	52.3	34.4	54.8	54.3				
Dose per scan^**^(ml)	9.7 (0.5–45)	8.1 (0.6–20)	N/A	N/A				
Cumulative dose per patient^**^ (mmol Gd)	7.5 (0.3–59.0)	7.6 (0.3–35.3)	N/A	N/A				

^a^Indicates significant differences within the contrast/non-contrast cohort

^b^Indicates significant difference between the contrast and non-contrast cohort

^**^Data are given as mean ± standard deviation and (range)

For contrast scans performed on adults, eGFR values were calculated from serum creatinine at the time point closest prior to MRI and used to stratify scans accordingly; stages 1/2 (eGFR ≥ 60 ml/min/1.73 m^2^, n = 19,168), stage 3 (30 ml/min/1.73 m^2^ ≤ eGFR < 60 ml/min/1.73 m^2^, n = 2,570), stage 4 (15 ml/min/1.73 m^2^ ≤ eGFR < 30 ml/min/1.73 m^2^, n = 464) and stage 5 (eGFR < 15 ml/min/1.73 m^2^, n = 123). Scan data for paediatric patients was stratified into two renal function categories: normal (n = 520) and impaired (n = 52) dependent upon interdepartmental age and gender-modified serum creatinine reference values.

Similar stratification by age and renal function categories was performed for the control data of non-contrast examinations.

### Hypersensitivity reactions

Hypersensitivity reactions were identified by any records of admission to A&E within a day of contrast-enhanced MRI with presenting symptoms that matched known reactions to gadoteric acid as given in the prescribing information and/or any general hypersensitivity reaction (Table 2).

**Table 2:** List of known adverse events (AEs) to gadoteric acid and other potential AEs as reported in the complete Accident & Emergency (A&E) dataset that was used to identify appropriate A&E admission records

	Presenting symptom
Known gadoteric acid AE	Allergic rash, Angioedema, Anaphylaxis, Anxiety, Arrhythmia, Asthma attack, Bradycardia, Bronchospasm, Burning sensation, Cardiac arrest, Coma, Conjunctivitis, Convulsion, Cramp, Cyanosis, Diarrhoea, Dizziness, Drowsiness, Erythema, Erythroderma, Exacerbation of asthma, Extravasation, Eyelid oedema, Fatigue, Fever, Headache, Hyperhidrosis, Hypertension, Hypotension, Injection site coldness, Injection site pain, Itching, Lacrimation, Laryngospasm, Malaise, Muscle contracture, Muscle spasm, Muscle weakness, Nausea, Ocular hyperaemia, Oropharyngeal discomfort, Pain in extremity, Palpitations, Paraesthesia, Parsomia, Pharyngeal oedema, Pins and needles, Pruritus, Rash, Salivary hypersecretion, Serum creatinine increase, Sleepiness, Superficial phlebitis, Syncope, Tachycardia, Tremor, Urticaria, Vomiting
Other potential AE identified as reported in A&E dataset	Acute renal failure, Anxiety state, Cardiac arrhythmia, Chronic renal failure, Dermatitis, Erythema nodosum, Exacerbation of asthma, Migraine headache, Needlestick, Needlestick wound, Panic attack, Primary hypertension, Purpura, Pyrexia, Rash, Sinus bradycardia, Sinus tachycardia, Situational collapse, Supra ventricular tachycardia, Urticarial rash, Ventricular tachycardia

### Potential toxicity signs

A search was performed on the dermatology records between 1 January 2004 and 30 November 2016 with the aim of identifying a recorded NSF diagnosis following any contrast-enhanced MRI scan. Chronic pain, a symptom sometimes associated with both NSF and GDD, was determined by the onset of new and regular prescriptions for chronic pain medication (British National Formulary Chapters 4.7.3 and 10.1.1) within 7 days post-MRI. Cases of PC-AKI were defined as an increase in serum creatinine greater than 25% from baseline within 3 days post-MRI in accordance with European Society of Urogenital Radiology (ESUR) guidelines [14] that were in place when this study was conducted.

### Statistical analysis

Statistical analysis was performed in SPSS statistical package (version 21.0, IBM SPSS). Statistical diagnostics were first performed to ensure the appropriate tests were used. Mann-Whitney and ANOVA tests with post-hoc analysis were performed to compare cohort demographics. Chi-squared tests were used to compare differences in rates of new chronic pain medication prescriptions between cohorts. ANCOVA testing was used to analyse changes in serum creatinine for both contrast and non-contrast scans across eGFR categories. Chi square and Fisher’s exact tests were used to compare the incidence of PC-AKI. A *p*-value of ≤ 0.05 was considered statistically significant.

## Results

### Study cohort – adults

A total of 37,813 contrast and non-contrast scans performed on a cohort of 21,770 adult patients were identified for analysis. 22,325 scans were contrast-enhanced, of which 14% were performed while the patient was deemed renally insufficient.

The average age at scan of all patients was significantly different between renal function categories. Age tended to increase with increasing RI (stages 1/2, 53.3 ± 15.4 years; stage 3, 69.6 ± 11.6 years; stage 4, 73.1 ± 12.7 years), but the average age decreased in the stage 5 category (60.5 ± 15.3 years). The average age of patients in stages 1/2 and 3 undergoing contrast-enhanced scans was higher when compared to those undergoing non-contrast scans (*p* < 0.0005), but this trend did not continue into stage 4 or 5 (*p* = 0.805 and *p* = 0.060, respectively). There were no statistically significant gender biases across renal function categories observed within the contrast group. For the non-contrast cohort, there was only a higher proportion of females than males in the stage 3 category (*p* < 0.0005). When comparisons were made between those having received contrast and those not, a significantly greater proportion of females underwent non-contrast scans in stages 1/2 and 3 than males (*p* < 0.0005).

### Hypersensitivity reactions

Two adult patients (0.01%) were admitted to A&E with hypersensitivity reactions within a day of contrast-enhanced MRI. The first patient presented with a headache that occurred following their first contrast-enhanced MRI. The patient underwent no further scans. The second patient presented in an anxiety state following their first contrast-enhanced MRI but did go on to undergo two further contrast-enhanced scans with no reactions.

### NSF

With an average follow-up time of 6.0 ± 2.5 years (range, 8 months–15 years), zero cases of NSF were diagnosed following the 3,157 gadoteric acid-enhanced MRI performed on patients with RI.

### PC-AKI

**Fig. 1:**
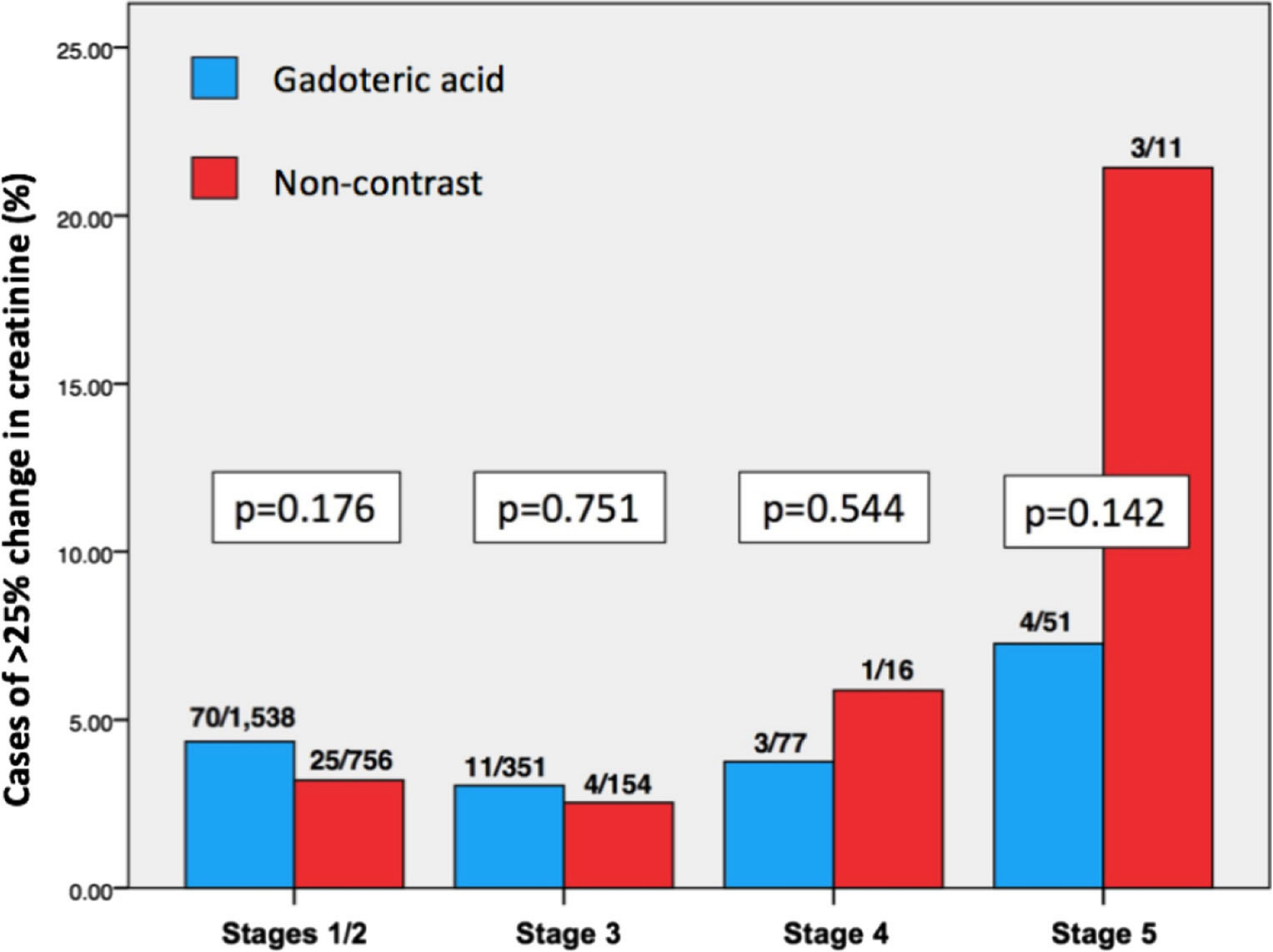
Observed rate of cases with > 25% increase in creatinine in adults depending on renal function and whether contrast was administered or not. Numbers above bars represent the number of cases with > 25% increase in creatinine/total number of scans investigated

Serum creatinine levels within 3 days pre- and post-MRI were available and percentage changes thus calculated for 2,105 contrast and 970 non-contrast scans. After controlling for the dependence of creatinine changes on eGFR category, there was no statistical difference in percentage changes of creatinine between contrast or non-contrast scans (-0.40% vs. 0.61%, *p* = 0.273). When this analysis was limited to only those scans performed on patients with renal function categorised as stage 4 or 5, those undergoing a non-contrast scan experienced an average serum creatinine increase of 7.31%, while the serum creatinine of those undergoing contrast scans decreased by an average of -4.40% (*p* = 0.023). There was no statistical difference in cases of a greater than 25% increase in serum creatinine post-MRI observed (Fig. 1) regardless of renal function or whether contrast was administered or not (*p* = 0.566).

### GDD

A total of ten (0.026%) cases of new and regular chronic pain medication prescriptions were noted following either contrast or non-contrast MRI. Seven of these were contrast-enhanced MRI. All patients had normal renal function at the time of scan. The rate was not statistically higher regardless of renal function or whether contrast was administered (*p* = 0.777).

### Paediatrics

A total of 1,265 scans performed on a cohort of 698 paediatric patients were identified for analysis. 572 scans were contrast-enhanced, of which 9% were performed while the patient was deemed renally insufficient.

Within both the normal and RI categories, the average age at scan was statistically higher in those undergoing contrast scans compared with non-contrast scans (*p* < 0.0005 and *p* = 0.015, respectively). There was no significant difference in gender proportions (*p* = 0.180) regardless of renal function or contrast group.

Zero paediatric patients were admitted to A&E with hyper-sensitivity reactions within a day of contrast enhanced MRI. After monitoring for an average time of 6.2 ± 2.4 years (range, 1–10 years), there have been zero cases of NSF diagnosed following 52 contrast-enhanced MRIs performed on 32 paediatric patients with RI. Percentage changes in serum creatinine did not differ significantly between the contrast and non-contrast group (0.32% vs. -6.05%, *p* = 0.113) and there was no significantly higher rate of increases over 25% observed (*p* = 0.841) regardless of renal function or contrast administration. One scan out of 1,265 (0.08%) was followed by an onset of a new, regular chronic pain medication prescription. At the time of the contrast-enhanced scan, the patient had normal renal function. The incidence of prescriptions for chronic pain medicine did not differ significantly across renal function or contrast group (*p* = 0.578).

## Discussion

‘Gadolinium toxicity’ is a generic term that had been ascribed to a myriad of signs and symptoms resulting from exposure to GdCAs, some of which have been confirmed – hypersensitivity reactions and NSF, whilst others are more debatable – PC-AKI and chronic pain. After having been deemed at an increased risk of gadolinium toxicity, the administration of GdCAs to patients with renal insufficiency has declined. Contrast-enhanced MRI scans may still be performed in this population if the benefit of information to be gained outweighs the potential risk, thus investigations may still continue retrospectively.

Through health data linkage, the anonymised patient data from 39,078 MRI scans, performed locally between 2004 and 2016, was linked with patient-specific electronic health records. Gadoteric acid was administered during 22,897 of these scans, 3,209 of which were performed on RI individuals. A low number of hypersensitivity reactions were observed within our adult cohort (0.01%) and none within our paediatric patients post-contrast exposure. After following these patients via their health records for an average of 6 years, zero cases of NSF were identified. Serum creatinine levels for the study cohort were largely unaffected (Fig. 1), with no statistically higher rate of PC-AKI following exposure to gadoteric acid when compared to non-contrast examinations, regardless of baseline renal function. Across all patients, there has been no significantly higher rate of chronic pain onset, a symptom that has been associated with GDD, when compared with patients who had undergone non-contrast scans.

To the best of our knowledge, this study is one of the largest and longest investigations into NSF diagnoses in cohorts with RI following GdCA administration. It is amongst the first to investigate for an array of potential signs and symptoms of gadolinium toxicity. No study has previously used data linkage in this manner to identify hypersensitivity reactions to gadoteric acid. Traditional adverse event monitoring methods have estimated reaction rates to gadoteric acid at between 0.12% [29] and 0.97% [30]. Rates of NSF and PC-AKI have been researched previously, with which this study agrees well. In particular, no unconfounded cases of NSF have been confirmed following gadoteric acid administration [2, 29, 31] and no significantly higher rates of PC-AKI were observed between the cohort of patients with RI exposed to gadoteric acid and those who underwent non-contrast scans only [31]. As GDD is a recently hypothesised clinical entity, few studies have investigated for potential symptoms. Whilst this study has investigated for the incidence of chronic pain onset, similar data linkage methodologies have been used to assess for cerebellar sequelae [32, 33]. In particular, Perrotta et al failed to observe the occurrence of clinical cerebellar syndrome in their gadoteric acid-exposed population [32].

Conversely, other GdCAs have been associated with some form of gadolinium toxicity. Although the vast majority of NSF cases have been seen following the administration of linear agents, there have been unconfounded cases reported with other macrocyclic agents [12, 34, 35] . In cases of suspected GDD, chronic pain is a frequent symptom [25] for which chronic pain medication has been administered in order to ease symptoms [26]. Available data on GdCA-induced PC-AKI varies widely, and with small sample sizes it is difficult to draw definitive conclusions [36], but PC-AKI has been observed following administrations of both gadodiamide [37] and gadopentetate dimeglumine [38].

Demonstrating that GdCAs can be used safely in this population may improve outcomes for patients with RI. By being able to examine patients using the full capabilities of MRI, co-morbidities and complications may be better diagnosed and treated [39]. Although an MRI-based clinical trial is likely to be deemed unethical at this time, health data linkage has allowed MRI-based research to continue in this cohort and further help to identify any potential clinical consequences of gadolinium toxicity. It was noted in the EMA’s report [12] that, regardless of renal function at the time of GdCA administration, it remains unknown whether later declines in patient health may trigger gadolinium toxicity symptoms. Thus, it is imperative to employ a surveillance technique that can accurately monitor patient populations over long periods of time. Ramahlo et al also suggests that reactions to gadolinium may depend somewhat on genetics [7]. As such, there is a potential for further studies to use health data linkage, incorporating genetic data, where available, to help develop understanding of how this disease may progress, if at all.

There are limitations to this study. Regarding the collection of hypersensitivity reaction data, it is possible that by only examining A&E records some mild adverse events may have been overlooked. Patients who suffer a mild reaction may present to their GP or other healthcare providers (e.g. community pharmacy) and, although there is potential for these records to also be examined, they were not available for this study. As NSF awareness increased within the time period of this study, precautionary methods, such as immediate post-MRI dialysis, may have been employed thus reducing any potential effects. The retrospective nature of this study allowed for NSF diagnoses to be determined only via dermatology records. It is appreciated, however, that NSF is a multi-factorial pathology and a definitive diagnosis includes clinical, biochemical and histopathological features [40], which were unavailable. Serum creatinine changes may have resulted from other factors that were not controlled for, such as cardiovascular disease, renal artery stenosis, advanced age, dehydration and use of nephrotoxic drugs. Averaging serum creatinine levels at precise time points pre- and post-MRI would have provided a clearer indication of renal function; however, these data were not available. Following the completion of this study, it was noted that new guidelines regarding PC-AKI were published [41] in which the definition of PC-AKI was amended. As access to the data had expired, the analysis could not be repeated using the new definitions. Unfortunately, height data were not recorded and thus the Schwartz formula could not be used to categorise paediatric renal function. Cohort demography differences are unlikely to have impacted results as gadolinium toxicity has been shown to have no age, gender or racial predisposition [11, 42]. It cannot be guaranteed that patients were administered no other GdCAs, nor sought medical attention in other institutions. In some cases, patients may not have consulted their general practitioners or sought any other form of medical attention. Beyond NSF, a disease profile relating to gadolinium deposition is unconfirmed so these results are merely conjecture. Chronic pain and PC-AKI have, however, all been associated with the administration of GdCAs, and using large datasets such as this one may be the only way to identify unconfounded symptoms, if any, caused by the agents.

Cases of potential gadolinium toxicity to gadoteric acid (Dotarem®) were investigated through the electronic linkage of health data. None of the 22,897 contrast-enhanced scans performed on adult and paediatric patients, including the 3,209 scans performed on patients with renal insufficiency, resulted in a diagnosis of NSF. A low number of hypersensitivity reactions post-contrast were observed. There was no statistically higher rate of chronic pain or cases of PC-AKI observed between the contrast- and non-contrast-enhanced scans investigated.

## Abbreviations

A&E: Accident & Emergency
EMA: European Medicines Agency
ESUR: European Society of Urogenital Radiology
GdCA: Gadolinium-based contrast agent
GDD: Gadolinium deposition disease
GP: General practitioner
MRI: Magnetic resonance imaging
NSF: Nephrogenic systemic fibrosis
PC-AKI: Post-contrast acute kidney injury
RI: Renal insufficiency
